# FATP1 Exerts Variable Effects on Adipogenic Differentiation and Proliferation in Cells Derived From Muscle and Adipose Tissue

**DOI:** 10.3389/fvets.2022.904879

**Published:** 2022-07-11

**Authors:** Jieping Huang, Duo Guo, Ruirui Zhu, Ye Feng, Ruirui Li, Xintong Yang, Deshun Shi

**Affiliations:** State Key Laboratory for Conservation and Utilization of Subtropical Agro-Bioresources, Guangxi University, Nanning, China

**Keywords:** intramuscular fat deposition, FATP1, variable effects, muscle-derived cells, adipose-derived cells

## Abstract

In livestock, intramuscular adipose tissue is highly valued whereas adipose tissue in other depots is considered as waste. Thus, genetic factors that favor fat deposition in intramuscular compartments over that in other adipose depots are highly desirable in meat-producing animals. Fatty acid transport 1 (FATP1) has been demonstrated to promote cellular fatty acid uptake and metabolism; however, whether it also influences cellular lipid accumulation remains unclear. In the present study, we investigated the effects of FATP1 on the differentiation and proliferation of adipocytes in five types of cells derived from muscle and adipose tissue and estimated the effects of FATP1 on intramuscular fat (IMF) deposition. We showed that FATP1 is mainly expressed in heart and muscle tissue in buffaloes as well as cells undergoing adipogenic differentiation. Importantly, we found that FATP1 promoted the adipogenic differentiation of muscle-derived cells (buffalo myocytes and intramuscular preadipocytes and mouse C2C12 cells) but did not affect, or even inhibited, that of adipose-derived cells (buffalo subcutaneous preadipocytes and mouse 3T3-L1 cells, respectively). Correspondingly, our results further indicated that FATP1 promotes IMF deposition in mice *in vivo*. Meanwhile, FATP1 was found to enhance the proliferative activity of all the assessed cells, except murine 3T3-L1 cells. These results provide new insights into the potential effects of FATP1 on IMF deposition, especially regarding its positive effects on meat quality in buffaloes and other livestock.

## Introduction

In livestock production, fat accumulated in subcutaneous and visceral depots is considered as waste. However, fat accumulated in muscle tissue (intramuscular fat [IMF]) is essential for improving the flavor and juiciness of meat, especially beef (1, 2). Therefore, strategies that enhance fat accumulation in muscle tissue while inhibit that in adipose tissue are important in animal production.

Several decades of investigation into fat deposition have revealed hundreds of genes that participate in this complex biological process (3, 4). Several of these genes, such as thyroid hormone responsive (*THRSP*) (5, 6), peroxisome proliferator-activated receptor gamma (*PPARG*) (7), and phosphoenolpyruvate carboxykinase 1 (*PCK1*) (8, 9), have been found to promote IMF deposition; however, they also favor the deposition of fat in other depots, such as subcutaneous and visceral fat depots.

Fatty acid transport 1 (FATP1), also known as solute carrier family 27 member 1 (SLC27A1), is known to enhance the transportation of fatty acids (FAs) (10) as well as their metabolism, including esterification and oxidation (11, 12). Accordingly, FATP1 has been suggested to affect lipid accumulation in cells and tissues. However, disagreements remain regarding the role of FATP1 in this process (13), with some studies supporting that FATP1 promotes lipid accumulation (14, 15) and others suggesting that FATP1 inhibits lipid accumulation (16, 17).

In this study, the effects of FATP1 on adipogenic differentiation and the proliferation of cells derived from muscle and adipose tissue were investigated in both buffaloes and mice. The results suggested that FATP1 promotes adipogenic differentiation in muscle-derived cells and enhances IMF deposition *in vivo*; in contrast, FATP1 does not affect, or even inhibits, fat deposition in adipose-derived cells. Our findings further indicated that FATP1 plays a positive role in the proliferation of all the assessed cells, except 3T3-L1 cells. These results provide a novel potential genetic factor for the improvement of meat quality in livestock and increase our knowledge of the variable effects of FATP1.

## Materials and Methods

### Ethics Statement

The buffaloes used in this study were bred for commercial use. All animal-based protocols were approved by the Institutional Animal Care and Use Committee at the College of Animal Science and Technology, Guangxi University. All efforts were made to minimize the suffering of the animals.

### Preparation of Buffalo Tissue Samples

Tissues from five organs—the heart, liver, spleen, lung, and kidney—as well as from the *longissimus dorsi* muscle and back fat were collected from Binlangjiang buffaloes (*n* = 6, 24 months old) (18) for gene expression profiling. All the tissues were sampled immediately after slaughter and frozen in liquid nitrogen. For primary cell isolation, *longissimus dorsi* muscle and back fat tissues were obtained from a buffalo slaughterhouse (Wufeng United Food Co., Ltd, Nanning, China). Freshly sampled tissues were kept in PBS supplemented with 1% penicillin/streptomycin and taken to the laboratory for cell isolation.

### RT-qPCR Analysis

Total RNA was isolated using TRIzol Reagent (Invitrogen, Carlsbad, CA, USA) according to the manufacturer's instructions and reverse-transcribed using the PrimeScript RT Reagent Kit with gDNA Eraser (TaKaRa, Dalian, China). Two-step qPCR was performed using SYBR Green I (TaKaRa) following the manufacturer's protocol. The expression levels of target genes were normalized to that of β-actin in 3T3-L1 and C2C12 cells and those of the β-actin and *GAPDH* genes in buffalo cells and tissues, respectively. Details of the primers used are provided in Supplementary Table 1. The cycle threshold (2^−ΔΔCt^) method was used to calculate the relative expression levels of candidate genes. Three replicates were run per sample.

### Cell Preparation and Culture

Primary myocytes and primary intramuscular preadipocytes were isolated from *longissimus dorsi* muscle tissue of buffaloes using enzymatic digestion (9). Buffalo primary subcutaneous preadipocytes were isolated from back fat tissue using the tissue block method as previously described (19). 3T3-L1 and C2C12 cells were purchased from ATCC (Shanghai, China) and cultured in DMEM (Gibco) supplemented with 10% FBS (Gibco) and 1% penicillin/streptomycin (Gibco) in 5% CO_2_ at 37 °C.

### Adenovirus Production

The overexpression of buffalo FATP1 (Reference Sequence: XM_006057903.2) and PPARG (Reference Sequence: MN867675.1) was achieved using an adenovirus system. Adenovirus production was undertaken by Hanbio Biotechnology Co., Ltd. (Shanghai, China). Briefly, the coding sequence (CDS) of a candidate gene was inserted into the AdMax system, which included a backbone plasmid (pHBGloxΔE1, 3cre) and a shuttle plasmid (pHBAd-EF1α-MCS-CMV-EGFP). EGFP was used as a visual indicator of the efficiency of transduction.

### Plasmid Construction and siRNA Oligonucleotides Synthesis

The overexpression of mouse *FATP1* (Reference Sequence: NM_011977.4) and *PPARG* (Reference Sequence: NM_011146.3) was achieved using the pcDNA3.1(+) plasmid. The CDS of each candidate gene was amplified from cDNA derived from C2C12 cells and inserted into the pcDNA3.1(+) vector (HindIII and XhoI restriction sites). Details of the primers used for plasmid construction are shown in Supplementary Table 1. siRNA oligonucleotides for mouse *FATP1* (GCACTGTACTAGTGCATAT) and negative control (β*-actin*) were purchased from Ruibo Biotechnology Co., Ltd, Guangzhou, China.

### Transduction and Transfection

For isolated cells, including myocytes, intramuscular preadipocytes, and subcutaneous preadipocytes, the adenovirus delivery system was utilized to achieve efficient overexpression. Cells were seeded in 12-well plates at 70% density. At 80% confluence, transduction was performed at the indicated multiplicity of infection (MOI) in myocytes and intramuscular preadipocytes and at 80% of the indicated MOI in subcutaneous preadipocytes.

For 3T3-L1 and C2C12 cells, overexpression and interference were achieved using the pcDNA3.1(+) plasmid and siRNA, respectively. Cells were seeded in 12-well plates at 50% density and were transfected at 70% confluence using Lipofectamine 3000 (Invitrogen) following the manufacturer's instructions.

### Induction of Adipogenic Differentiation

Two days after transduction or transfection, cells were treated with inducing medium containing 10 μg/mL insulin, 1 μ? dexamethasone, 0.5 mM IBMX, and 1 μ? rosiglitazone for 2 days and subsequently treated with maintenance medium containing 10 μg/mL insulin and 1 μM rosiglitazone until lipid droplets were clearly visible (within approximately 6–8 days). The four agents (insulin, dexamethasone, IBMX, and rosiglitazone) were purchased from Sigma (Milwaukee, WI, USA).

### Oil Red O Staining and Quantification

Following adipogenic differentiation, cells were stained with oil red O. For this, the cells were washed three times with PBS, fixed in 10% formalin first for 5 min and then for 1 h, washed with 60% isopropanol, stained with 0.3% oil red O (Sigma) for 20 min, and again washed three times with PBS. Finally, cells were observed and imaged under a microscope. For the quantitative analysis of lipid accumulation, lipid was eluted using 100% isopropanol, following which the absorbance at 510 nm was measured using a spectrophotometer.

### Triglyceride Detection

The triglyceride (TG) content of cells and tissues was detected using a TG quantification kit (Applygen Technologies Inc., Beijing, China) according to the manufacturer's protocol.

### EdU and CCK-8 Assays

The proliferation rate of cells was investigated using EdU and CCK-8 assays, which were, respectively performed using a Cell-Light EdU Apollo 567 *in vitro* Imaging Kit (RiboBio, Guangzhou, China) and a CCK-8 kit (Vazyme, Nanjing, China) according to the manufacturers' protocols. Both assays were performed 24 h after transduction or transfection.

### *In vivo* Experiments

The Kunbai mice (*n* = 9, approximately 10 weeks old) were purchased from Guangxi Medical University. The mice were randomly divided into three groups, namely, a negative control group (*n* = 3, injected with the pcDNA3.1 plasmid), a positive control group (*n* = 3, injected with the pcDNA3.1_PPARG plasmid), and the experimental group (*n* = 3, injected with the pcDNA3.1_FATP1 plasmid). A total of 20 μg of plasmid was mixed with 40 μL of Entranster *in vivo* transfection reagent (Engreen, Beijing, China) and left to stand for 15 min. The mixture was then injected into the left *gastrocnemius* muscle of each mouse according to the manufacturer's instructions. After 3 weeks, 50 μL of glycerol was injected into the same anatomical location of each animal. After 2 weeks, the animals were euthanized and the *gastrocnemius* muscle was harvested for gene expression profiling (RT-qPCR) and quantification of TG content.

### Statistical Analysis

Data were analyzed in SPSS software using one-way ANOVA. The Holm–Sidak method was used for correction for multiple comparisons. A *p*-value < 0.05 was considered significant. Data are presented as means ± SD by GraphPad Prism.

## Results

### The Expression Profile of FATP1 in Tissues and Cells During Adipogenic Differentiation

**Figure 1:**
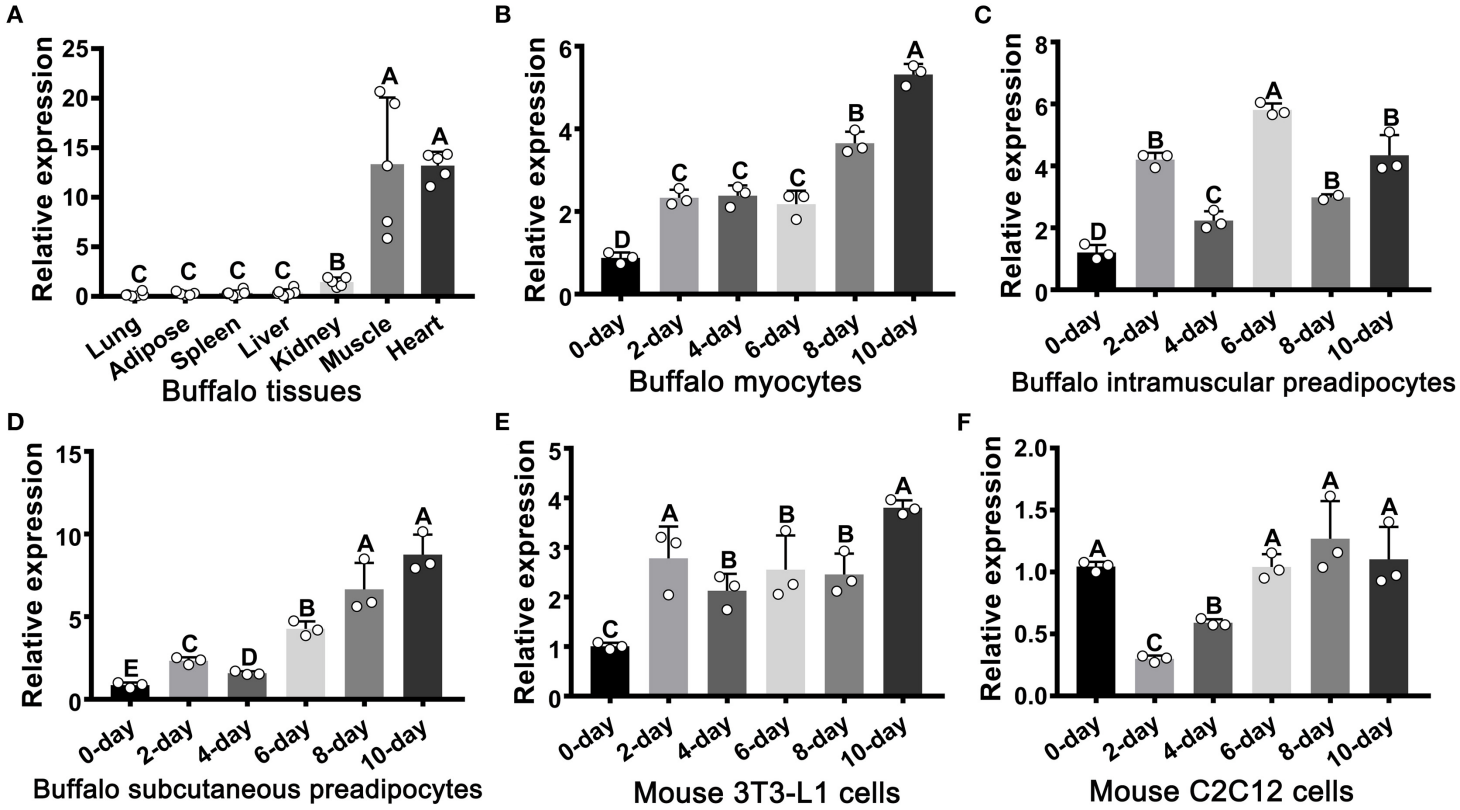
The mRNA expression profile of FATP1 in tissues and cells as determined by RT-qPCR. **(A)** The tissue expression profile of FATP1 in the buffalo. **(B–D)** Expression dynamics of FATP1 during adipogenic differentiation in buffalo myocytes, intramuscular preadipocytes, and subcutaneous preadipocytes. **(E,F)** Expression dynamics of FATP1 during adipogenic differentiation in mouse 3T3-L1 and C2C12 cells. Data are presented as means ± SD; lowercase letters indicate a significant difference (*p* < 0.05), uppercase letters indicate a highly significant difference (*p* < 0.01).

The expression of FATP1 was analyzed across seven tissues in the buffalo (Figure 1A). As expected, FATP1 was mainly expressed in cardiac and skeletal muscle (Figure 1A). In buffalo myocytes, FATP1 expression was markedly upregulated during adipogenic trans-differentiation (Figure 1B). In buffalo intramuscular and subcutaneous preadipocytes, FATP1 expression was also upregulated during adipogenic differentiation, although to a varying extent (Figures 1C,D), and a similar FATP1 expression profile was observed during the adipogenic differentiation of mouse 3T3-L1 preadipocytes (Figure 1E). However, during the adipogenic trans-differentiation of mouse C2C12 cells, the expression of FATP1 was first downregulated and then returned to the original level (Figure 1F). The results showed that, overall, FATP1 was highly expressed in cells during adipogenic differentiation. The adipogenic differentiation of all cell types assessed was monitored using the adipogenic markers *PPARG* and *CEBPA* (Supplementary Figure 1).

### FATP1 Promotes Lipid Accumulation in Buffalo Myocytes

**Figure 2:**
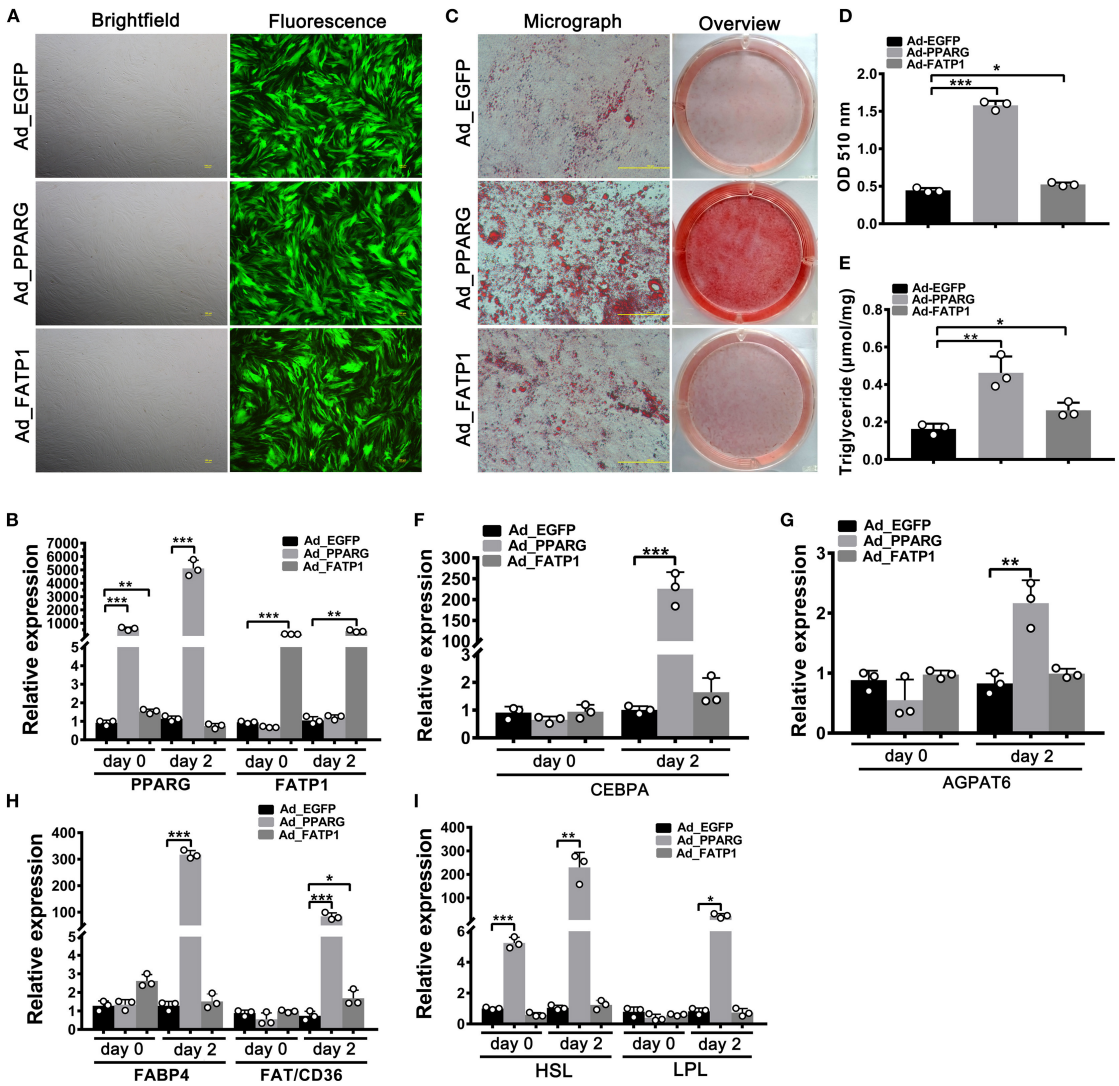
The overexpression of FATP1 promotes lipid accumulation in buffalo myocytes. **(A)** Micrographs of buffalo myocytes 2 days after adenovirus transduction under bright and fluorescent light. Scale bar, 100 μm. The mRNA expression levels of *PPARG* and *FATP1* **(B)**, the adipogenic gene *CEBPA* **(F)**, the lipogenic gene *AGPAT6* **(G)**, the fatty acid uptake-related genes *FABP4* and *FAT*/*CD36* **(H)**, and the lipogenesis genes *HSL* and *LPL* **(I)** in the control and experimental groups on day 0 and day 2 of adipogenic trans-differentiation (2 and 4 days after adenovirus transduction, respectively). **(C)** Oil red O staining of “myocytes” on day 8 of adipogenic trans-differentiation. Scale bar, 100 μm. **(D,E)** Quantification of lipid accumulation and triglyceride content in “myocytes” on day 8 of adipogenic trans-differentiation. EGFP overexpression (Ad_EGFP) served as a negative control; PPARG+EGFP overexpression (Ad_PPARG) served as a positive control; FATP1+EGFP overexpression (Ad_FATP1) served as the experimental group. Data are presented as means ± SD, * *p* < 0.05, ** *p* < 0.01, *** *p* < 0.001.

To evaluate the effects of FATP1 on lipid accumulation in buffalo myocytes, we overexpressed FATP1 by using an adenoviral system (Ad_FATP1) with EGFP serving as the indicator (Ad_EGFP). PPARG, the core regulator of adipogenesis, was employed as a positive control (Ad_PPARG). As shown in Figure 2A, the high intensity of green fluorescence indicated a high transduction efficiency. Both PPARG and FATP1 were significantly upregulated 2 days after transduction (on day 0 of adipogenic differentiation), with increases of ~500- and ~200-fold, respectively (Figure 2B). As expected, compared with the Ad_EGFP group, the lipid content was significantly increased in both of the Ad_PPARG and the Ad_FATP1 groups (Figures 2C,D). Similarly, the TG content was significantly increased in the Ad_PPARG and Ad_FATP1 groups compared with the Ad_EGFP group (Figure 2E). On day 2 of adipogenic differentiation, the levels of all the detected markers—the adipogenic gene *CEBPA* (Figure 2F), the lipogenic gene *AGPAT6* (Figure 2G), the FA uptake-related genes *FABP4* and *FAT*/*CD36* (Figure 2H), and the lipogenesis genes *HSL* and *LPL* (Figure 2I)—were upregulated in the Ad_PPARG group. In the Ad_FATP1 group, meanwhile, only the FA uptake-related gene *FAT*/*CD36* was upregulated, and only at a low level (Figure 2H). These data indicated that FATP1 promotes lipid accumulation in buffalo myocytes, although not to the same extent as PPARG.

### FATP1 Promotes Adipogenic Differentiation in Buffalo Intramuscular Preadipocytes but Not in Subcutaneous Preadipocytes

**Figure 3:**
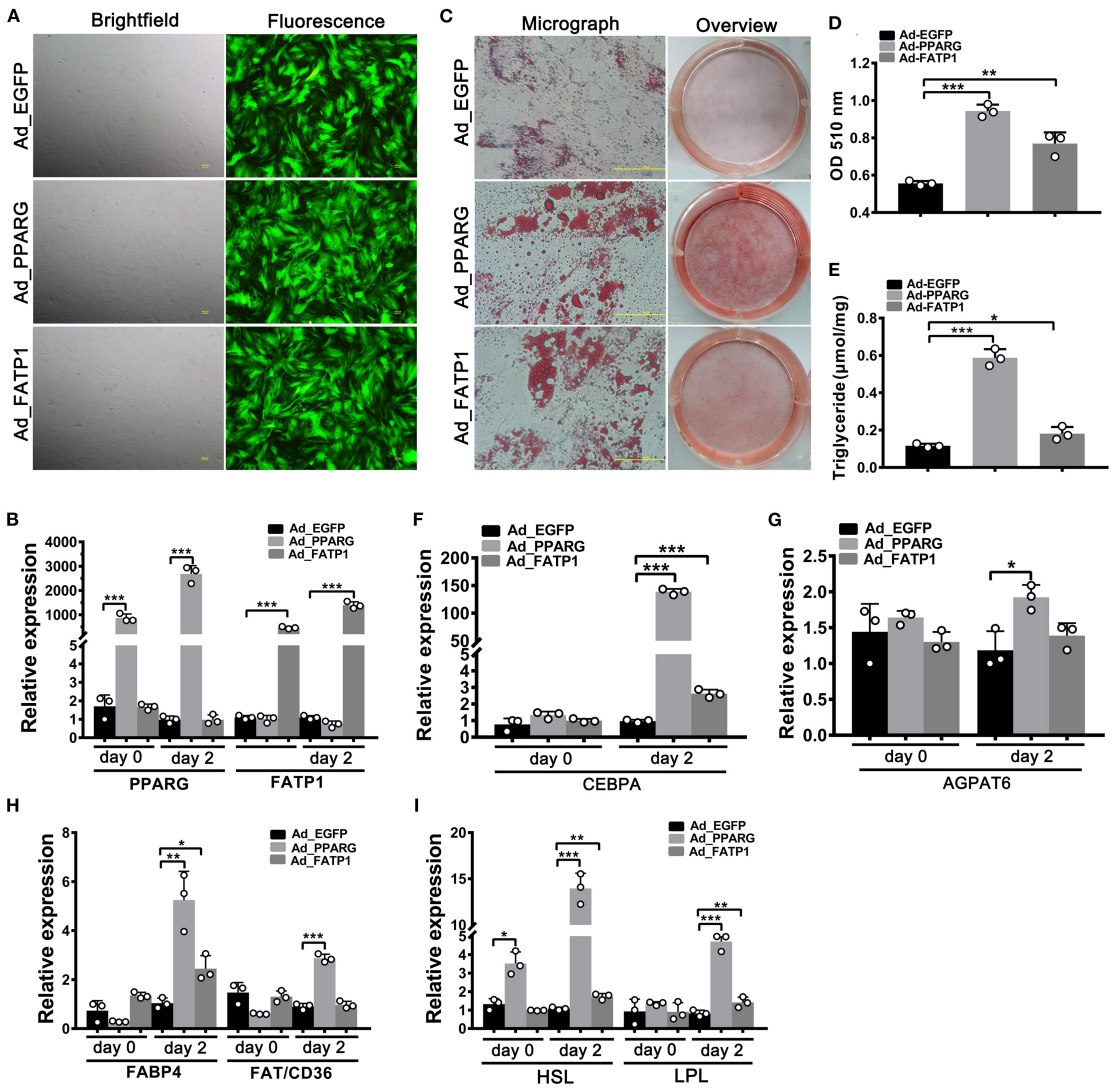
The overexpression of FATP1 promotes adipogenic differentiation in buffalo intramuscular preadipocytes. **(A)** Micrographs of buffalo myocytes on day 2 of adenovirus transduction under bright and fluorescent light. Scale bar, 100 μm. The mRNA expression levels of *PPARG* and *FATP1* **(B)**, the adipogenic gene *CEBPA* **(F)**, the lipogenic gene *AGPAT6* **(G)**, the fatty acid uptake-related genes *FABP4* and *FAT*/*CD36* **(H)**, and the lipogenesis genes *HSL* and *LPL* **(I)** in the control and experimental groups on day 0 and day 2 of adipogenic trans-differentiation (2 and 4 days after adenovirus transduction, respectively). **(C)** Oil red O staining of “myocytes” on day 8 of adipogenic trans-differentiation. Scale bar, 100 μm. **(D,E)** Quantification of lipid accumulation and triglyceride content in “myocytes” on day 8 of adipogenic trans-differentiation. EGFP overexpression (Ad_EGFP) served as a negative control; PPARG+EGFP overexpression (Ad_PPARG) served as a positive control; FATP1+EGFP overexpression (Ad_FATP1) served as the experimental group. Data are presented as means ± SD, * *p* < 0.05, ** *p* < 0.01, *** *p* < 0.001.

Studies have indicated that the effects of FATP1 on lipid accumulation can vary according to cell type (13). Accordingly, we next investigated the effects of FATP1 on adipogenic differentiation in buffalo intramuscular and subcutaneous preadipocytes using the same strategy as that used for buffalo myocytes. As shown in Figure 3A, high transduction efficiency was achieved in buffalo intramuscular preadipocytes. Both PPARG and FATP1 were significantly upregulated 2 days after transduction (on day 0 of adipogenic differentiation), with increases of ~800- and ~400-fold, respectively (Figure 3B). Importantly, lipid accumulation and TG content in the Ad_PPARG and Ad_FATP1 groups were significantly higher than those in the Ad_EGFP group (Figures 3C–E). On day 2 of adipogenic differentiation, the levels of all the detected markers were upregulated in the Ad_PPARG group (Figures 3F–I), while in the Ad_FATP1 group, the adipogenic gene *CEBPA* (Figure 3F), the FA uptake-related gene *FABP4* (Figure 3H), and the lipogenesis genes *HSL* and *LPL* (Figure 3I) were upregulated. These results indicated that FATP1 promotes the adipogenic differentiation in buffalo intramuscular preadipocytes.

**Figure 4:**
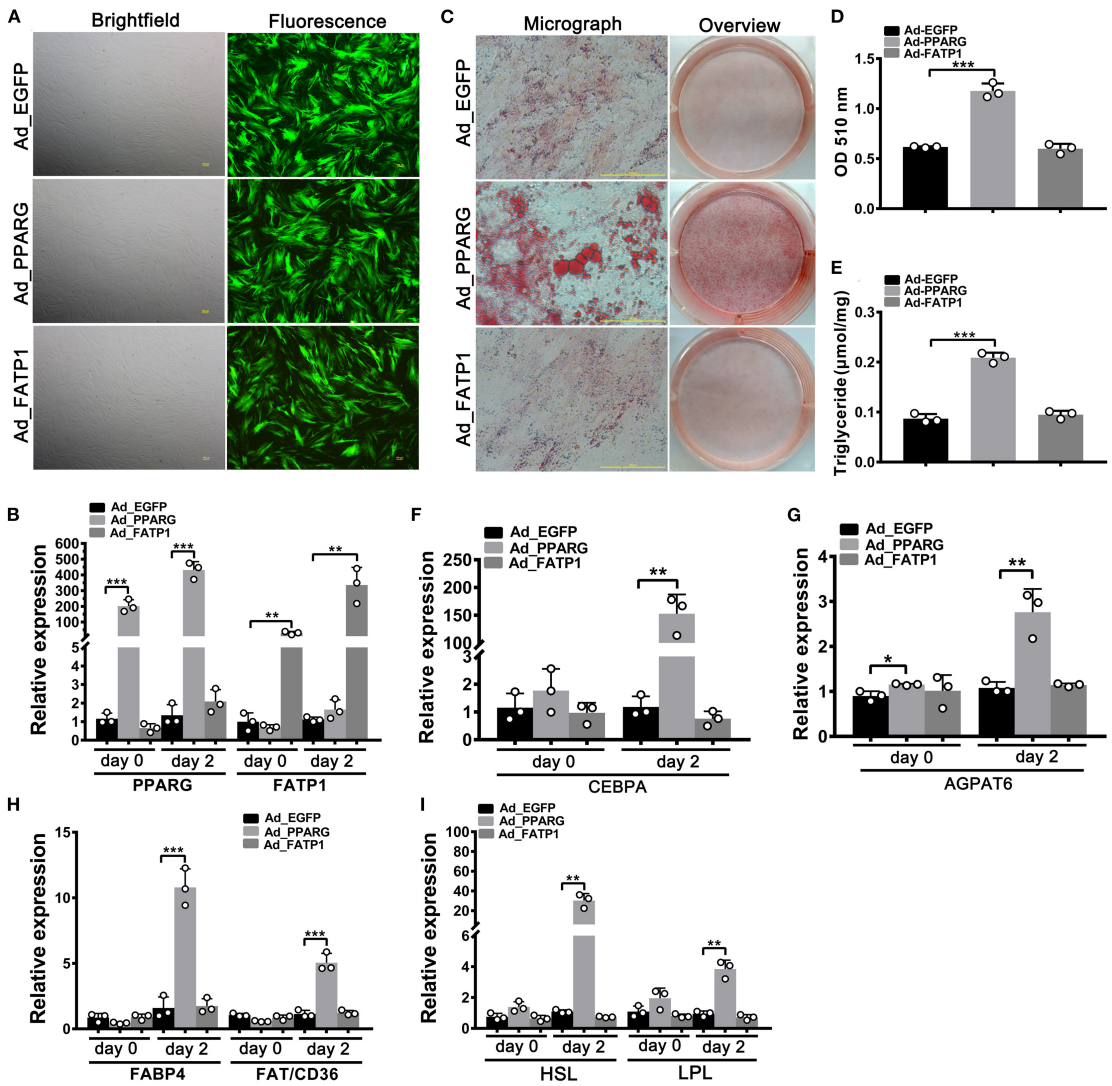
The overexpression of FATP1 does not affect adipogenic differentiation in buffalo subcutaneous preadipocytes. **(A)** Micrographs of buffalo myocytes on day 2 of adenovirus transduction under bright and fluorescent light. Scale bar, 100 μm. The mRNA expression levels of *PPARG* and *FATP1* **(B)**, the adipogenic gene *CEBPA* **(F)**, the lipogenic gene *AGPAT6* **(G)**, the fatty acid uptake-related genes *FABP4* and *FAT*/*CD36* **(H)**, and the lipogenesis genes *HSL* and *LPL* **(I)** in the control and experimental groups on day 0 and day 2 of adipogenic trans-differentiation (2 and 4 days after adenovirus transduction, respectively). **(C)** Oil red O staining of “myocytes” on day 8 of adipogenic trans-differentiation. Scale bar, 100 μm. **(D,E)** Quantification of lipid accumulation and triglyceride content in “myocytes” on day 8 of adipogenic trans-differentiation. EGFP overexpression (Ad_EGFP) served as a negative control; PPARG+EGFP overexpression (Ad_PPARG) served as a positive control; FATP1+EGFP overexpression (Ad_FATP1) served as the experimental group. Data are presented as means ± SD, * *p* < 0.05, ** *p* < 0.01, *** *p* < 0.001.

A high overexpression efficiency was obtained in subcutaneous preadipocytes as well (Figures 4A,B). Lipid accumulation (Figures 4C,D) and TG content (Figure 4E) were increased in the Ad_PPARG group relative to that in the Ad_EGFP group; however, no significant difference was detected between the Ad_FATP1 and Ad_EGFP groups (Figures 4C,D). Additionally, compared with the Ad_EGFP group, the levels of all the adipogenic markers were upregulated in the Ad_PPARG group, but not in the Ad_FATP1 group (Figures 4F–I). These findings suggested that FATP1 does not affect the adipogenic differentiation in buffalo subcutaneous preadipocytes.

### FATP1 Promotes the Proliferation of Buffalo Myocytes, Intramuscular Preadipocytes, and Subcutaneous Preadipocytes

**Figure 5:**
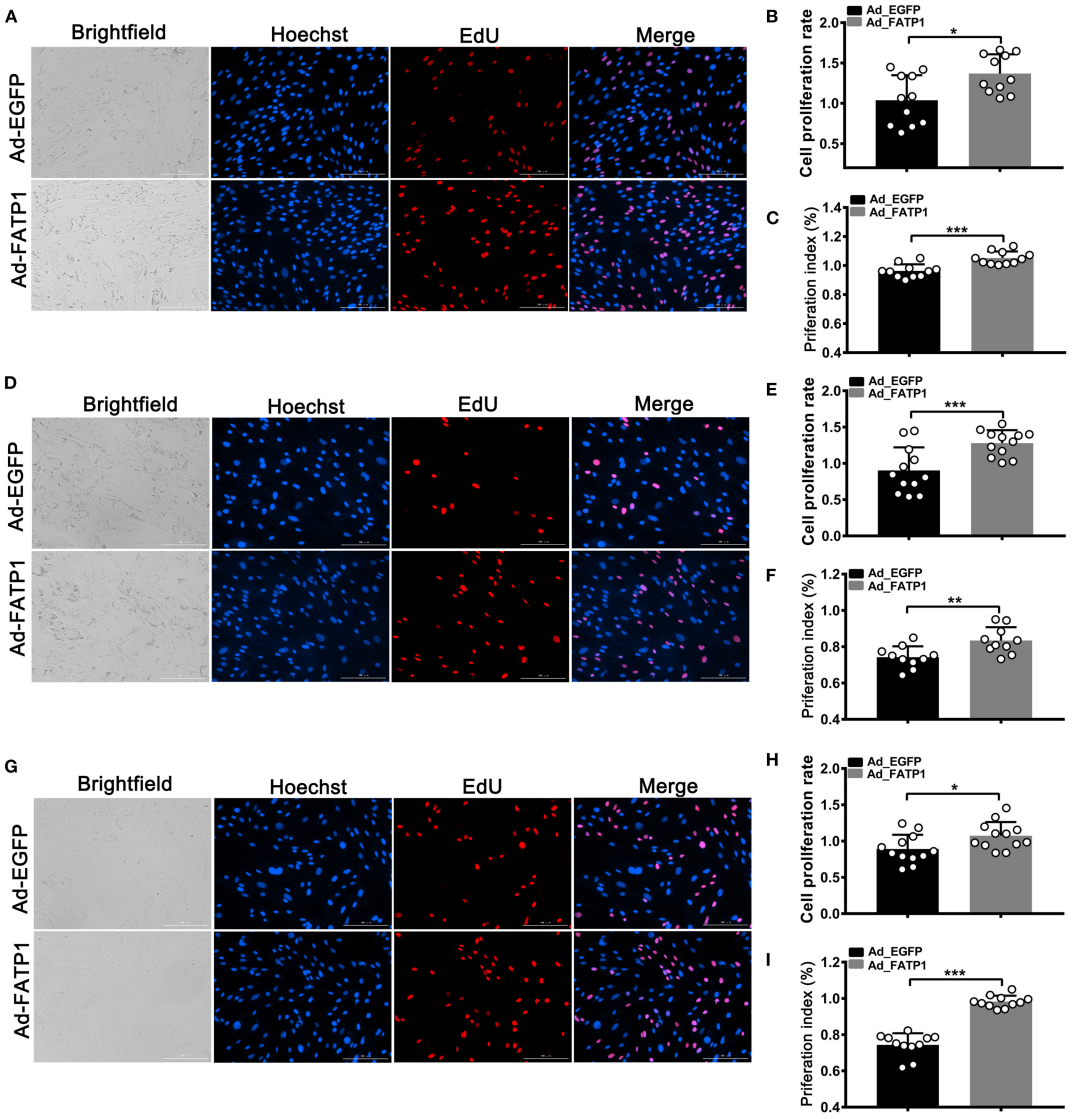
FATP1 promotes the proliferation of buffalo myocytes, intramuscular preadipocytes, and subcutaneous preadipocytes. **(A,D,G)** Micrographs of EdU assays for buffalo myocytes, intramuscular preadipocytes, and subcutaneous preadipocytes, respectively. Blue indicates the nuclei of all the cells; EdU-stained nuclei are shown in red. Scale bar, 200 μm. **(B,E,H)** Statistical analysis of EdU assays for buffalo myocytes, intramuscular preadipocytes, and subcutaneous preadipocytes, respectively. **(C,F,I)** Statistical analysis of CCK-8 assays for buffalo myocytes, intramuscular preadipocytes, and subcutaneous preadipocytes, respectively. Data are presented as means ± SD; * *p* < 0.05, ** *p* < 0.01, *** *p* < 0.001.

EdU and CCK-8 assays were employed to uncover the effects of FATP1 on the proliferative ability of buffalo myocytes, intramuscular preadipocytes, and subcutaneous preadipocytes. In myocytes, the cell proliferation rate in the Ad_FATP1 group was significantly higher than that in the control group (Ad_EGFP) as evidenced by EdU staining (*p* < 0.05; Figures 5A,B). Correspondingly, the results of the CCK-8 assay indicated that the cell proliferation index in the Ad_FATP1 group was significantly higher than that in the Ad_EGFP group (*p* < 0.001; Figure 5C). Similar results were obtained for intramuscular preadipocytes and subcutaneous preadipocytes (Figures 5D–I). These findings demonstrated that FATP1 enhances the proliferative capacity of buffalo myocytes, intramuscular preadipocytes, and subcutaneous preadipocytes.

### FATP1 Promotes Both Adipogenic Differentiation and Proliferative Activity in C2C12 Cells

**Figure 6:**
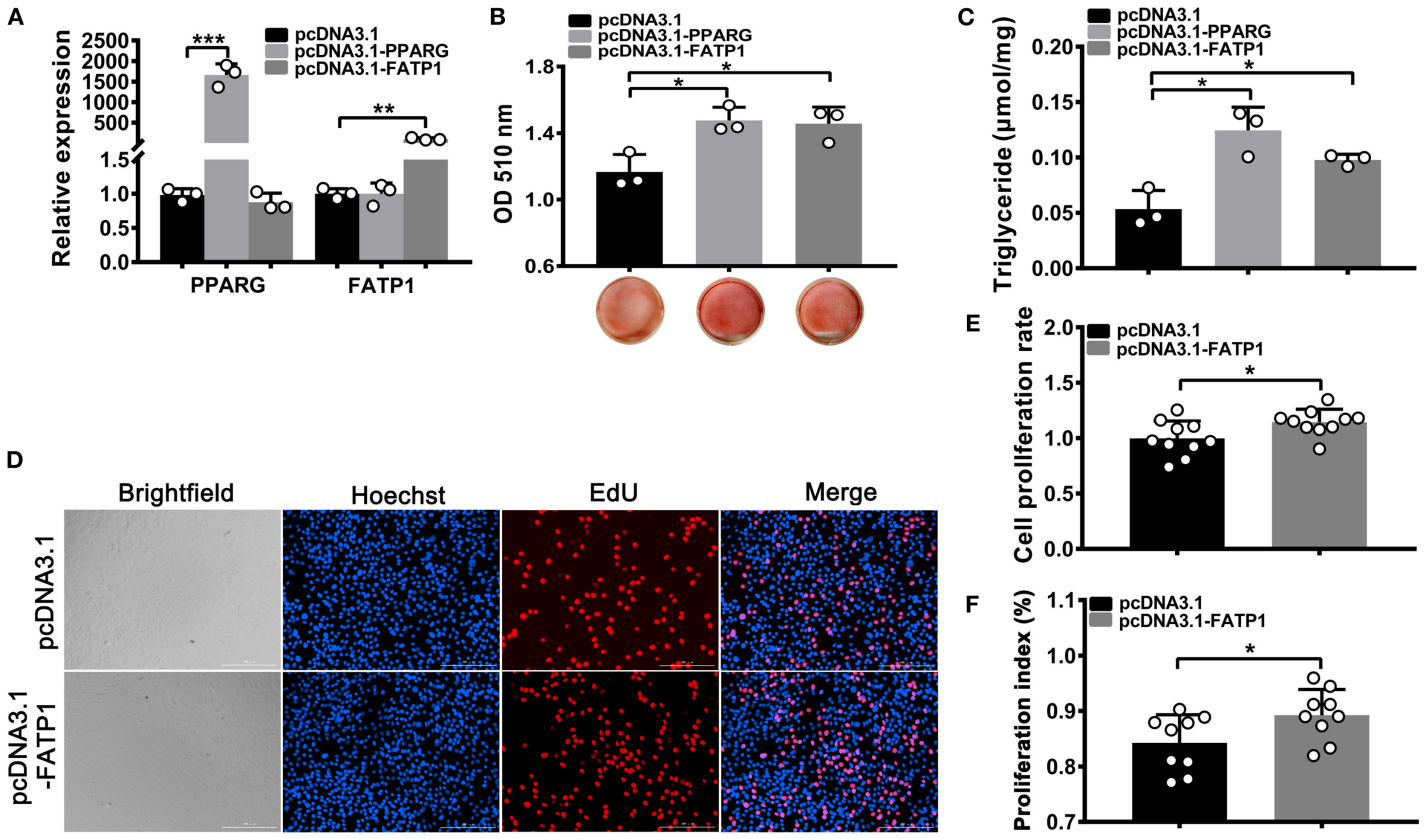
FATP1 enhances the proliferation and promotes the adipogenic trans-differentiation of C2C12 cells. **(A)** The mRNA expression levels of PPARG and FATP1 as determined by RT-qPCR. **(B)** Histogram showing the quantitation of oil red O staining. **(C)** The triglyceride levels of C2C12 cells on day 8 of adipogenic trans-differentiation. **(D)** Micrographs of the EdU assay in C2C12 cells. Scale bar, 200 μm. **(E)** Statistical analysis of EdU assays in C2C12 cells. **(F)** Statistical analysis of CCK-8 assays in C2C12 cells. Data are presented as means ± SD, * *p* < 0.05, ** *p* < 0.01, *** *p* < 0.001.

To evaluate the effects of FATP1 on lipid accumulation and proliferation in mouse C2C12 cells, the CDS of FATP1 was cloned into the pcDNA3.1(+) vector (pcDNA3.1_FATP1) and transfected into C2C12 cells. PPARG (pcDNA3.1_PPARG) was used as a positive control. High efficiency of overexpression was achieved, with ~1500-fold and ~100-fold increases in expression for PPARG and FATP1, respectively, being observed (Figure 6A). Importantly, both lipid accumulation (*p* < 0.05; Figure 6B) and TG content (*p* < 0.05; Figure 6C) were increased in both of the PPARG and FATP1 groups compared with that in the negative control group. The cell proliferation rate was significantly increased in the FATP1 group compared with that in the negative control group (*p* < 0.05; Figures 6D–F).

### FATP1 Inhibits Both Adipogenic Differentiation and Proliferative Ability in 3T3-L1 Cells

**Figure 7:**
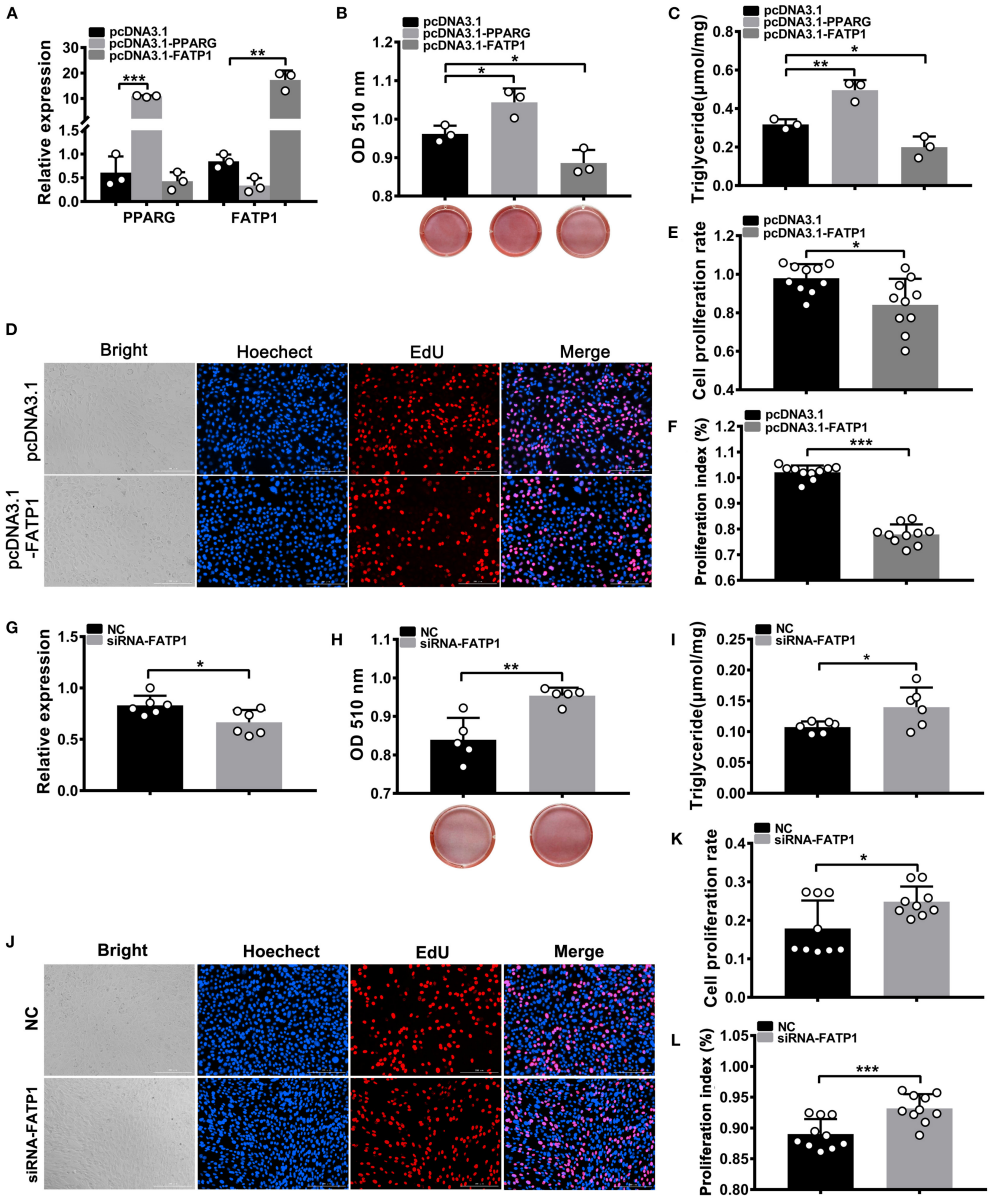
FATP1 inhibits both the proliferation and adipogenic differentiation of 3T3-L1 cells. **(A)** The overexpression efficiency of PPARG and FATP1 and **(G)** the interference efficiency in 3T3-L1 cells determined by RT-qPCR. Histogram showing the quantitation of oil red O staining after FATP1 overexpression **(B)** and FATP1 interference **(H)**. The triglyceride levels on day 8 of adipogenic differentiation in 3T3-L1 cells after FATP1 overexpression **(C)** and FATPT interference **(I)**. Micrographs of EdU assays in 3T3-L1 cells after FATP1 overexpression **(D)** and FATP1 interference **(J)**. Scale bar, 200 μm. Statistical analysis of EdU assays in 3T3-L1 cells after FATP1 overexpression **(E)** and FATP1 interference **(K)**. Statistical analysis of CCK-8 assays in 3T3-L1 cells after FATP1 overexpression **(F)** and FATP1 interference **(L)**. Data are presented as means ± SD, * *p* < 0.05, ** *p* < 0.01, *** *p* < 0.001.

To test whether the effects of FATP1 on adipogenic differentiation and proliferation differed between C2C12 and 3T3-L1 cells, we overexpressed FATP1 and PPARG in 3T3-L1 cells using the same system as that used for C2C12 cells. We found that PPARG and FATP1 levels were increased by only ~10- and ~15-fold, respectively, compared with the controls (Figure 7A). Moreover, relative to that in the control group, lipid accumulation was significantly decreased in the FATP1 overexpression group and significantly increased in the PPARG overexpression group (*p* < 0.05; Figure 7B). Similar results were obtained for TG content (*p* < 0.05; Figure 7C). However, cell proliferation was inhibited in the FATP1 overexpression group (Figures 7D–F), which was not consistent with that in buffalo subcutaneous preadipocytes. Thus, further interference of FATP1 was accomplished by siRNA to confirm the effects of FATP1 on lipid accumulation and proliferation in mouse 3T3-L1 cells. As shown in Figures 7G–I, interference of FATP1 enhanced lipid accumulation and TG synthesis. Besides, interference of FATP1 promoted the proliferation of 3T3-L1 cells as well (Figures 7J–L). Therefore, the results of overexpression of FATP1 were consistent well with those of interference. FATP1 inhibited both adipogenic differentiation and proliferative ability in 3T3-L1 cells.

### FATP1 Increases Intramuscular Fat Content *in vivo*

**Figure 8:**
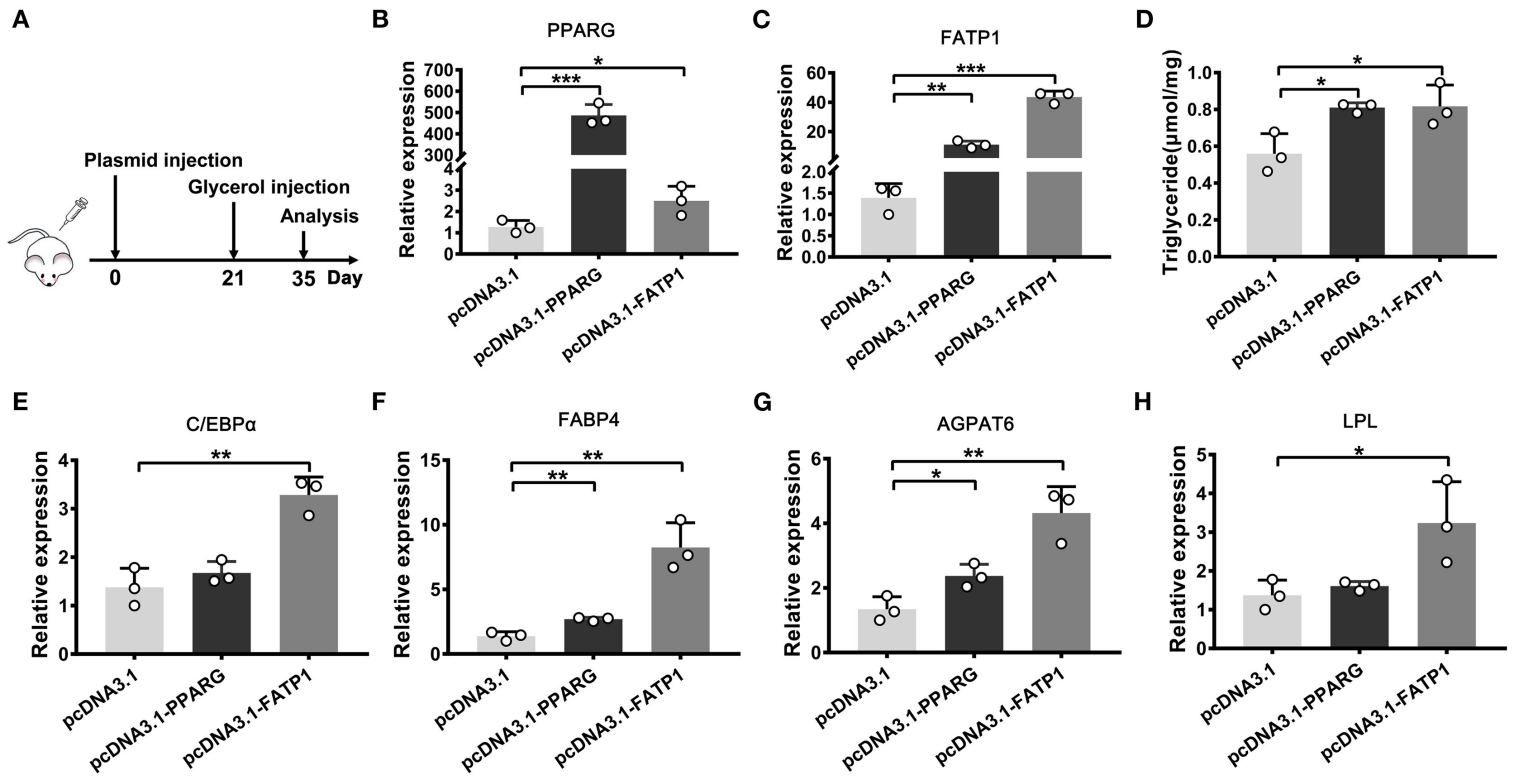
The overexpression of FATP1 increases the intramuscular fat content in the mouse. **(A)** The strategy employed for intramuscular plasmid injection. The expression of *PPARG* **(B)**, *FATP1* **(C)**, *CEBPA* **(E)**, *FABP4* **(F)**, *AGPAT6* **(G)**, and *LPL* **(H,D)** triglyceride levels in the *gastrocnemius* muscle injected with the indicated plasmid 2 weeks after glycerol injection. Data are presented as means ± SD, * *p* < 0.05, ** *p* < 0.01, *** *p* < 0.001.

The above evaluation in cellular level indicated that FATP1 is functional conservative in cell adipogenic differentiation between buffaloes and mice. To demonstrate the effects of FATP1 on IMF *in vivo*, we utilized the glycerol model of muscle regeneration in which IMF deposition is more easily observed (20). We injected the FATP1_expressing plasmid (pcDNA3.1_FATP1) and the PPARG_expressing plasmid (pcDNA3.1_PPARG) into the *gastrocnemius* muscle of mice using Entranster *in vivo* transfection reagent (21). The *in vivo* overexpression strategy *i*s presented in Figure 8A. Both PPARG (positive control) and FATP1 were significantly overexpressed (Figures 8B,C). Importantly, the TG content was significantly increased in FATP1_ and PPARG_overexpressing mice compared with that in mice administered the control plasmid (*p* < 0.05; Figure 8D). Correspondingly, at the molecular level, the levels of the adipogenic marker genes *CEBPA* and *FABP4*, the fat synthesis-related gene *AGPAT6*, and the lipolysis gene *LPL* were significantly upregulated in animals overexpressing either FATP1 or PPARG (Figures 8E–H). These results indicated that FATP1 increases IMF deposition *in vivo* in mice.

## Discussion

In this study, we found that FATP1 exerts variable effects on adipogenic and proliferative potential of cells derived from muscle and adipose tissue as well as IMF deposition. The main results of the present study indicated that (1) in buffaloes, FATP1 is mainly expressed in cardiac and skeletal muscle and is highly expressed in cells undergoing adipogenic differentiation; (2) FATP1 promotes adipogenic differentiation in cells derived from muscle tissue but does not affect, or even inhibits, adipogenic differentiation in adipose tissue-derived cells; (3) FATP1 enhances the proliferative capacity of buffalo myocytes, intramuscular preadipocytes, and subcutaneous preadipocytes as well as that of murine C2C12 cells, but inhibits the proliferation of 3T3-L1 cells; (4) FATP1 enhances IMF deposition in the mouse *in vivo*.

FATP1 is a key transporter in cellular FA uptake (22, 23). Tissues such as heart, skeletal muscle, and adipose tissue display rapid FA uptake and metabolism. In mice, FATP1 mRNA is abundantly detected in the heart, skeletal muscle, and adipose tissue (24), while in humans, FATP1 mRNA is highly expressed in skeletal muscle and adipose tissue (25). Recently, it was reported that bovine FATP1 mRNA is mainly detected in the heart, skeletal muscle, and adipose tissues (26). In the present study, FATP1 mRNA was mainly detected in heart and skeletal muscle, whereas its expression level was relatively low in adipose tissue (Figure 1A). Indeed, it has been reported that bovine FATP1 mRNA was abundantly expressed in heart and skeletal muscle but not in adipose tissue (10). Thus, results from different studies can vary, likely due to individual differences in metabolic rates, which can be affected by both genetic and environmental factors. This idea is supported by the variable FATP1 expression profile observed during the adipogenic differentiation process in cells with different origins (Figures 1B–F). Nevertheless, our data showed that FATP1 mRNA was mainly upregulated and exhibited high levels of expression in cells undergoing adipogenic differentiation (Figures 1B–F). These results support that FATP1 is vital for cellular FA uptake and metabolism.

Given its important role in uptake, esterification, and oxidation of FAs, FATP1 has been proposed to function as a regulator of lipid accumulation in cells. However, the effects of FATP1 on cellular lipid accumulation remains unclear. While some studies have demonstrated that FATP1 promotes lipid accumulation in muscle-derived cells such as quail QM-7 muscle cells (17), human muscle cells (27), and porcine intramuscular preadipocytes (15), others have reported that FATP1 does not affect, or even inhibits, lipid accumulation in muscle tissue (16, 28, 29). Here, our data support that FATP1 promotes lipid accumulation in muscle-derived cells, including mouse C2C12 cells (Figures 6A–C), buffalo myocytes (Figure 2), and buffalo intramuscular preadipocytes (Figure 3), and that FATP1 enhances IMF deposition in the mouse *in vivo* (Figure 8). We further found that FATP1 inhibits adipogenic differentiation in 3T3-L1 cells (Figure 7), which is consistent with previously reported results (30); however, FATP1 did not affect adipogenic differentiation in buffalo subcutaneous preadipocytes (Figure 4). In summary, FATP1 promotes adipogenesis in muscle tissue and muscle tissue-derived cells, but does not influence, and may even inhibit, adipogenesis in cells derived from adipose tissue.

Results of several studies have indicated that specific molecules or genes can exert differential effects on adipogenesis in intramuscular and subcutaneous adipocytes (31–33). Indeed, IMF is significantly different from subcutaneous and visceral fat (34, 35). Muscle cells and mature adipocytes secrete specific factors to further regulate fat deposition (36). Accordingly, the gene regulatory networks involved in the development of intramuscular and subcutaneous adipocytes are different (37, 38). In livestock, especially bovines, IMF is highly appreciated for its significant positive effect on meat quality, while fat deposition in other compartments, such as subcutaneous or visceral depots, is considered as waste (39). Regulatory factors that favor IMF deposition over subcutaneous or visceral fat deposition are highly desirable in the production of livestock animals. Our results indicate that FATP1 may fulfill this criterion. We demonstrate that FATP1 promotes IMF deposition in the mouse *in vivo* (Figure 8). As previous mentioned, FATP1 is a key transporter in cellular FAs uptake (22, 23). Overexpression of FATP1 significantly enhances FAs uptake in muscle. The excess FAs can be used for TG synthesis in the cytoplasm or delivered into mitochondria for ATP produce (40). When the efficiency of TG synthesis higher than that of ATP produce, lipid droplets can be accumulated in muscle tissue. On the other hand, though FATP1 has been demonstrated to be with no effect or negative effect on lipid accumulation in adipose tissue-derived cells, further *in vivo* experiment is necessary.

Cell proliferation underlies tissue development. Both proliferation and adipogenic differentiation require a large amount of energy. Since FATP1 plays an important role in adipogenic differentiation, it could also affect the proliferation of cells. Our data demonstrated that FATP1 promotes the proliferation of cells derived from muscle and adipose tissues in buffalo (Figure 5). Thus, FATP1 promotes the development of muscle and adipose tissues by enhancing cell proliferation. In livestock, the development of muscle and adipose tissue comprises two main stages, namely, cell proliferation in the early stage and differentiation in the adult stage (39, 41, 42). Thus, in the early stages, high levels of FATP1 in muscle benefits muscle development; concomitantly, low levels of FATP1 in adipose tissue will reduce waste in buffalo production. Our data also showed that FATP1 promotes the proliferation of C2C12 cells but inhibits that in 3T3-L1 preadipocytes (Figures 7D–F), indicating that the effects of FATP1 on the cell proliferation are different between buffaloes and mice. Therefore, using mice as animal models to evaluate the effects of FATP1 on cell proliferation may need further consideration.

In conclusion, our findings support that FATP1 enhances fat deposition in intramuscular compartments but does not affect, or even inhibits, that in other depots. Our data further indicate that FATP1 enhances the proliferative ability of both muscle- and adipose-derived cells, except 3T3-L1 cells. These results provide new insights into the potential effects of FATP1 on animal production, especially regarding its role in improving meat quality in buffaloes and other livestock.

## Data Availability Statement

The datasets presented in this study can be found in online repositories. The names of the repository/repositories and accession number(s) can be found in the article/supplementary materials. The datasets have been deposited in figshare and the accession number (DOI) is 10.6084/m9.figshare.20174003.

## Ethics Statement

The animal study was reviewed and approved by Institutional Animal Care and Use Committee at the College of Animal Science and Technology, Guangxi University. Written informed consent was obtained from the owners for the participation of their animals in this study.

## Author Contributions

JH designed the study, analyzed the data, and drafted the manuscript. DG performed the experiments and analyzed the data. RZ, YF, RL, and XY helped to carry out the experiments and collected tissue samples. DS provided technical guidance and the experimental platform. All authors read and approved the final version of the manuscript.

## Funding

This work was supported by the National Natural Science Foundation of China (32060747 and U20A2051) and the Guangxi Natural Science Foundation (2020JJA130143 and 2021AC19014).

## Conflict of Interest

The authors declare that the research was conducted in the absence of any commercial or financial relationships that could be construed as a potential conflict of interest.

## Publisher's Note

All claims expressed in this article are solely those of the authors and do not necessarily represent those of their affiliated organizations, or those of the publisher, the editors and the reviewers. Any product that may be evaluated in this article, or claim that may be made by its manufacturer, is not guaranteed or endorsed by the publisher.
