# Supplementary material for: FATP1 Exerts Variable Effects on Adipogenic Differentiation and Proliferation in Cells Derived From Muscle and Adipose Tissue
Source: Front Vet Sci. 2022 Jul 11;9:904879. doi: 10.3389/fvets.2022.904879 (PMC9310014; doi:10.3389/fvets.2022.904879)
Supplement: Supplementary file 1 [file Table_1.DOCX]

Supplementary Material

# Supplementary Figures and Tables

## Supplementary Figures


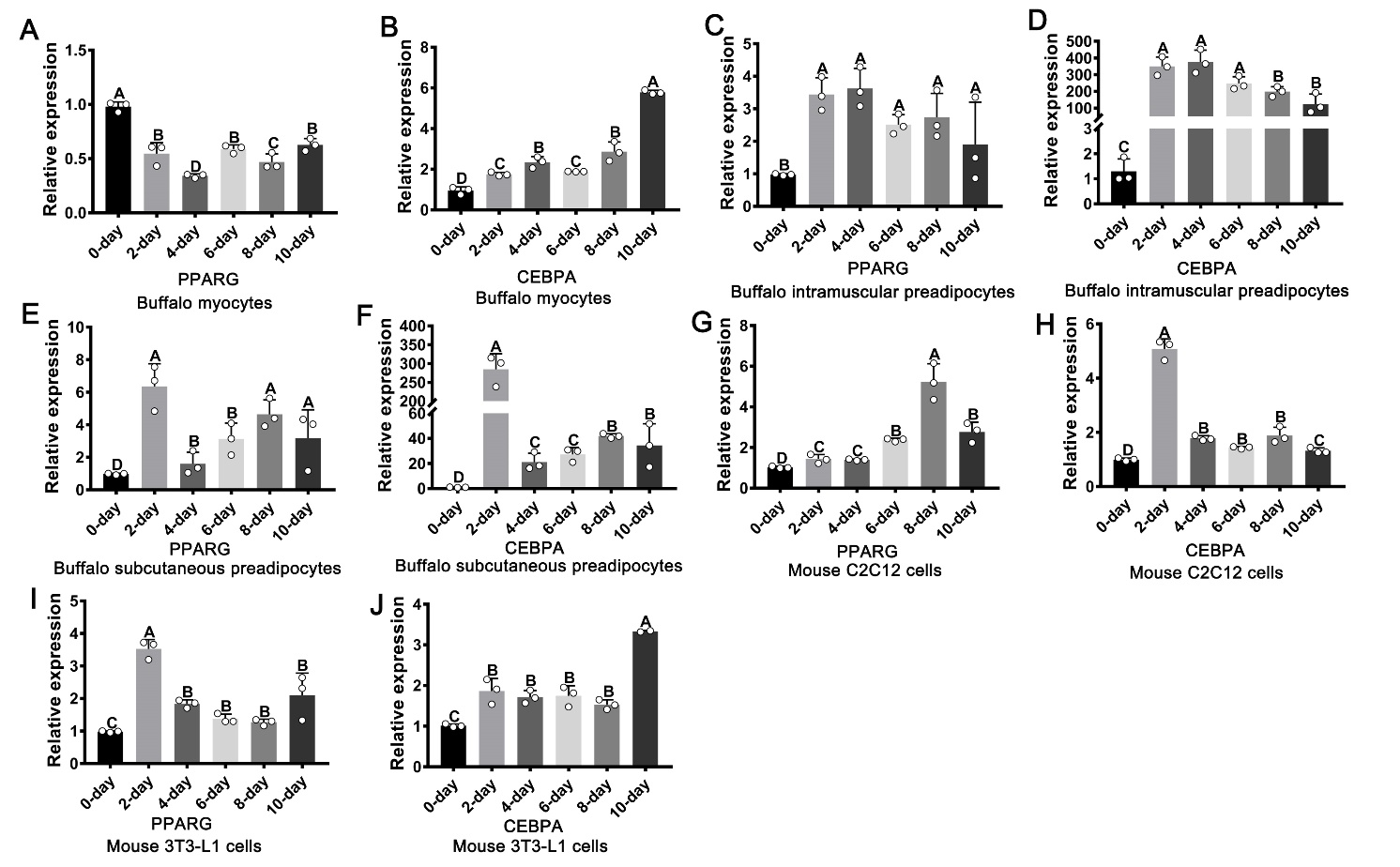


**Supplementary Figure 1.** The mRNA expression profile of PPARG and CEBPA during the adipogenic differentiation of buffalo myocytes, intramuscular adipocytes, and subcutaneous adipocytes and mouse C2C12 and 3T3-L1 cells. (**A, C, E, G, and I**) The expression level of PPARG during the adipogenic differentiation of buffalo myocytes, intramuscular adipocytes, and subcutaneous adipocytes and mouse C2C12 and 3T3-L1 cells. (**B, D, F, H, and J**) The expression level of CEBPA during the adipogenic differentiation of buffalo myocytes, intramuscular adipocytes, and subcutaneous adipocytes and mouse C2C12 and 3T3-L1 cells. Data are presented as means ± SD, * *p* < 0.05, ** *p* < 0.01, *** *p* < 0.001.

## Supplementary Tables

**Supplementary Table 1.** Details of primers used in the present study.

| Gene | Sense primer (5’–3’) | Antisense primer (5’–3’) | Production length (bp) | Used for |
| --- | --- | --- | --- | --- |
| β-actin (buffalo) | CATCCTGACCCTCAAGTA | CTCGTTGTAGAAGGTGTG | 91 | qRT-PCR |
| GAPDH (buffalo) | CACTCACTCTTCTACCTT | GCCAAATTCATTGTCGTA | 91 | qRT-PCR |
| FATP1 (buffalo) | TGGGAGGAGTTCACGGAGC | TTGAAGCCACAGGAACCGAC | 116 | qRT-PCR |
| PPARG (buffalo) | AGCCCAAGTTCGAGTTTGCT | TCTTGTATGTCCTCAATGGGCT | 139 | qRT-PCR |
| C/EBPα (buffalo) | GGTGCGTCTAAGATGAGGGG | GTAGGAATCGGAGCGGTGAG | 130 | qRT-PCR |
| FABP4 (buffalo) | AAGTCAAGAGCATCGTAA | CCAGCACCATCTTATCAT | 111 | qRT-PCR |
| CD36 (buffalo) | TGGAAAGGACGACATAAGCAAA | TGGAAATGAGGCTGCATCTGT | 118 | qRT-PCR |
| HSL (buffalo) | CAGTGTCCAAGACAGAGCCA | GCAGCTTCAGGCTTTTGAGG | 102 | qRT-PCR |
| LPL (buffalo) | GTCGCCGCAGACAGGATTAC | CTCAGCTGTGTCTTCGGGAG | 87 | qRT-PCR |
| AGPAT6 (buffalo) | CGATGACCAGACAGGCAGAG | TGTACAGCTTCTGCTGCTCC | 159 | qRT-PCR |
| DGAT1 (buffalo) | GGTCGCGGCCTTCGAT | TCTACGTCTCCGTCCTTGTCT | 118 | qRT-PCR |
| FATP1 (mouse) | GCAGCATTGCCAACATGGAC | GTGTCCTCATTGACCTTGACCAGA | 103 | qRT-PCR |
| PPARG (mouse) | GAGCTGACCCAATGGTTGCT | AAAAACCCTTGCATCCTTCACA | 234 | qRT-PCR |
| β-actin (mouse) | TTCCTTCTTGGGTATGGAAT | GAGCAATGATCTTGATCTTC | 203 | qRT-PCR |
| C/EBPα (mouse) | ACTCTTCACTAACGGCTGGG | ACCCTTGGACAACTAGGGGA | 142 | qRT-PCR |
| FABP4 (mouse) | CGACAGGAAGGTGAAGAGCATC | AACTCTTGTGGAAGTCACGCC | 154 | qRT-PCR |
| LPL (mouse) | GCGTAGTTCCAGCAGCAAAG | GAGAAATCTCTTCCCGCGTCT | 143 | qRT-PCR |
| AGPAT6 (mouse) | ATCAAGCCTCCTCTCCTTGT | ACAGCATCTTCATCTTTCTCTCG | 170 | qRT-PCR |
| FATP1-OE (mouse) | CCC*AAGCTT*ATGCGGGCTCCTGGAGCAG | CGC*GGATCC*CCAGGCTCAGAGTGAGAAGTCGC | 1947 | Construction of overexpression vector |
| PPARG-OE (mouse) | CCC*AAGCTT*ATGGGTGAAACTCTGGGAGATT | CGC*GGATCC*GGTGTCAGATTTTTTTCCCTCA | 1595 | Construction of overexpression vector |

Note: the restriction sites of primers are shown in italics.
